# Integrative Oncology for High-Grade Glioma: A Case Report on the Combined Effects of Oncothermia and Complementary Therapies

**DOI:** 10.7759/cureus.66492

**Published:** 2024-08-09

**Authors:** Pradeep MK Nair, Renganathan Ramalakshmi, Muniappan Devibala, Maruthanayagam Saranya, Sekar Sivaranjini, R Thangavelu, Manickam Mahalingam

**Affiliations:** 1 Department of Integrative Oncology, Mirakle Integrated Health Centre, Pollachi, IND; 2 Department of Modern Medicine, Mirakle Integrated Health Centre, Pollachi, IND

**Keywords:** complementary therapies, integrative medicine, cancer management, brain tumor treatment, quality of life

## Abstract

High-grade gliomas are aggressive brain tumors with a poor prognosis despite conventional treatments such as surgery, radiation, and chemotherapy. Integrative oncology, combining conventional and complementary therapies, may offer additional benefits in managing these complex cases. We present a 68-year-old male farmer diagnosed with high-grade glioma in the left medial temporal lobe. The patient presented with severe headache, disturbed sleep, and anxiety, and experienced an episode of fever and seizure. He refused conventional radiation therapy due to concerns about side effects and opted for an integrative medicine protocol. This protocol included oncothermia, high-dose vitamin C therapy, hydrogen inhalation, ozone therapy, magnet therapy, fasting, acupuncture, pulsed electromagnetic field therapy, yoga therapy, hydrotherapy, biologicals, and dietary modifications. The patient underwent 12 sessions of oncothermia over 24 days, combined with other integrative therapies. MRI scans before and after treatment showed a reduction in tumor size from 3.6 x 2.9 x 2.5 cm to 3.4 x 2.7 x 2.5 cm, corresponding to a 12% decrease in volume. Hematological parameters (complete blood count, liver function test, kidney function test, C-reactive protein), cancer markers (carcinoembryonic antigen, lactate dehydrogenase), and mental health indices (quality of life, survival rate) also showed significant improvement. The patient experienced no adverse events and reported enhanced quality of life. This case report suggests that an integrative oncology approach, combining oncothermia and various complementary therapies, may be an effective treatment option for high-grade gliomas, particularly for patients intolerant to conventional therapies. Further research, including randomized controlled trials, is necessary to validate these findings and determine the specific contributions of each therapy.

## Introduction

Gliomas are a diverse group of primary brain tumors arising from glial cells, with glioblastoma being the most common and aggressive subtype. These tumors present significant diagnostic and therapeutic challenges due to their rapid growth, infiltrative nature, and poor prognosis [1]. The incidence of high-grade gliomas (approximately six cases per 100,000 population), increases with age, and they are often associated with a variety of neurological symptoms that can mimic other conditions, complicating early diagnosis. Despite, the availability of standards of care like chemotherapy and radiation, the prognosis and survival rate of high-grade gliomas are very poor [2]. This implicates the need for newer technologies and supportive therapies that can improve the prognosis and survival rate.

In recent years, integrative oncology has emerged as a complementary approach in cancer care, combining conventional treatments with evidence-based complementary therapies to improve patient outcomes and quality of life. This approach is particularly relevant in the management of gliomas, where standard treatments like surgery, radiation, and chemotherapy are often insufficient to achieve long-term control.

In this case report, we present a 68-year-old male who was admitted with complaints of fever and a single episode of seizure. Initial clinical evaluation suggested an infectious or inflammatory process; however, subsequent imaging and histopathological studies revealed a high-grade glioma located in the left temporal lobe. This case explores the potential benefits of integrative oncology in managing high-grade gliomas. By incorporating integrative therapies, such as oncothermia, nutritional support, mind-body practices, and adjunctive use of biologicals, we aim to enhance the patient's quality of life and potentially improve clinical outcomes.

## Case presentation

**Figure 1:**
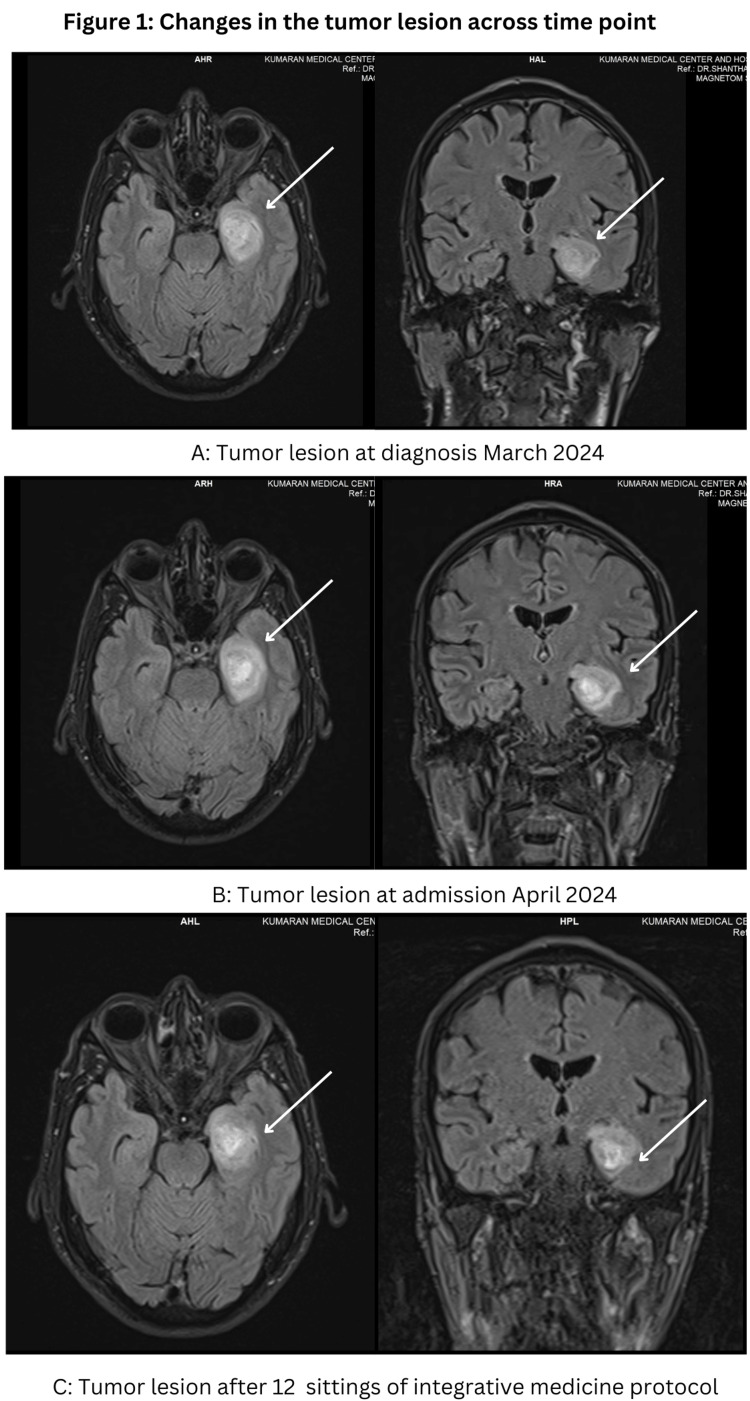
Changes in the tumor lesion over various time points. Images (A) and (B) show ill-defined mass lesions with internal necrotic areas, lobulated margins, and internal hemorrhagic areas in the left medial temporal lobe, involving uncus, amygdala, and head of the left hippocampus, measuring 3.6 x 2.9 x 2.5 cm. Image (C) shows T2/fluid-attenuated inversion recovery (FLAIR) heterointense peripherally enhancing lesion measuring 3.4 x 2.7 x 2.5 cm, seen in the left medial temporal lobe with mild surrounding edema.

The patient was a farmer who presented to our integrative medicine setting with complaints of severe headaches, disturbed sleep, and anxiety for two weeks. He was admitted to a multi-specialty hospital following an episode of high-grade fever and a seizure (a single episode for five minutes). Subsequently, he was diagnosed with high-grade glioma in March 2024 at a conventional oncology center, as his magnetic resonance imaging (MRI) indicated a ring-enhancing heterointense mass lesion (1.9 x 1.6 x 2.25 cm) in the left medial temporal lobe with mild surrounding edema (Figure 1). He was advised to undergo a biopsy and radiation therapy; however, he refused, citing concerns about the side effects associated with radiation therapy. He was taking levetiracetam (500 mg) daily, prescribed by the oncologist to prevent any further episodes of seizure.

The patient did not have any other comorbidities or functional disabilities. A positron emission tomography (PET) scan revealed a fluorodeoxyglucose (FDG)-avid peripherally enhancing lesion measuring 2.2 x 1.7 cm in the left medial temporal lobe (maximum standardized uptake value = 13.3) with mild perilesional edema. No other malignant lesions elsewhere in the body were noted. He was admitted as an inpatient in our integrative oncology facility in April 2024. An integrative medicine protocol was planned by a group of physicians, including medical experts from yoga and naturopathy, homeopathy, and medical oncology. The protocol included counseling, oncothermia (modulated electro-hyperthermia), intravenous high-dose vitamin C therapy, ozone therapy, hydrogen therapy, acupuncture, ethylenediaminetetraacetic acid (EDTA) chelation, yoga therapy, hydrotherapy, magnet therapy, Hydrosun therapy (Hydrosun Medizintechnik, Müllheim, Germany), biologicals, and diet therapy. The detailed protocol is outlined in Table 1.

**Table 1:** Integrative medicine protocol.

Intervention	Frequency of intervention
Counseling	Daily
Oncothermia (EHY-2030, Oncotherm, Budaörs, Hungary)	Alternate days (a total of 24 sessions was provided with a cooling period of 3 weeks in between each 12 sessions)
High-dose vitamin C therapy	2-3 times per week, the total dose not exceeding 90 grams per week
Ethylenediaminetetraacetic acid (EDTA) chelation	EDTA chelation was performed once in 7 days to remove heavy metal toxins
Ozone therapy	Daily (ozone is administered in the form of rectal insufflation/ear insufflation/saline ozone, and minor autohemotherapy)
Hydrogen inhalation (AT_HO-600 Hydrogen Inhalation Machine, Athena, Mumbai, India)	Daily inhalation of hydrogen for 30 minutes
Diet therapy	The patient was advised to follow a daily diet that is completely millet-based and free of sugar, refined wheat flour, and refined grains. This diet includes carrot, beetroot, and apple juice, as well as fresh vegetable salads and soups. Additionally, a time-restricted feeding schedule of 14:10 was also recommended
Liposomal curcumin therapy	Daily (5-10 ml liposomal curcumin), Mirakle, Coimbatore, India
Pulsed electromagnetic field (PEMF) therapy (PULSATRON at 10 Hz field frequency, Madras Institute of Magnetobiology, Chennai, India)	Daily for 30 minutes, exposed to PEMF using a controlled magnetic field assembly
Probiotic supplementation	Daily (two doses of Liv-Bio Alpha pre and probiotic supplements, Liv Bio Pharma, Poovarany, India)
Co-Q enzyme therapy	Daily (5-15 ml liposomal Co-Q 10 enzyme), Mirakle, Coimbatore, India
Coffee enema	Once a week (three tablespoons of chicory-free coffee is boiled in one liter of water, allowed to cool down, filtered, and then 500 ml is administered through the rectum in the form of an enema)
Hydrosun (Hydrosun 750, Hydrosun Medizintechnik, Müllheim, Germany)	Daily for 20 minutes (Hydrosun light therapy)
Acupuncture	Scalp acupuncture (Jiao Shun-fa School) - motor control area. Other acupuncture points like the large intestine (4), stomach (36), spleen (6), and back - Shu points and Ah-shi points were punctured daily for 30 minutes for 15 days
Yoga therapy	Daily 30-45 minutes (includes pranayama, meditation, and asanas)
Enema	Once a week to relieve constipation
Hot foot bath	As an when there is fatigue and headache
High-dose vitamin D supplementation	Once a week (60000 IU after monitoring the levels of parathyroid hormones and ionized calcium levels)
Injection dexamethasone	8 mg/day administered intravenously for one week to relieve edema
Injection N-acetyl cysteine	200 mg once in a week administered intravenously

The outcomes of the therapies were assessed through an MRI of the brain taken before and after the session. Furthermore, changes in hematological markers were observed through a complete blood count, liver function test, kidney function test, C-reactive protein, cancer markers such as carcinoembryonic antigen, D-dimer, ferritin, and lactate dehydrogenase, glycemic indices such as glycosylated hemoglobin, and vitamin D levels. Additionally, we used the Edmonton Symptom Assessment Scale (ESAS) [3] to measure changes in symptoms, the European Organization for Research and Treatment of Cancer Quality of Life Questionnaire (EORTC QLQ-C30) to measure quality of life [4], and the Chuang Prognostic Scale to predict survival [5].

The patient was treated as an inpatient and underwent 12 sessions of oncothermia on alternate days, along with other therapies as reported in Table 1, for a total duration of 24 days from April 2024 to May 2024. No adverse events or worsening of symptoms were reported during the treatment. An MRI repeated two weeks after the completion of the treatment revealed a reduction in the size of the lesion from 3.6 x 2.9 x 2.5 cm to 3.4 x 2.7 x 2.5 cm, which corresponds to an approximate 12% decrease in volume (Figure 1). Furthermore, there was a significant improvement in his hematological parameters, cancer markers, and mental health indices (Tables 2-4). The results are tabulated in Table 2. The patient is currently being followed up weekly with one session of oncothermia per week and other integrative medicine therapies for 12 weeks. He is presently stable and symptom-free.

**Table 2:** Changes in complete blood count, cancer markers, and anthropometric profile. RBC: red blood cells (million/μl); HB: hemoglobin (g/dl); PCV: packed cell volume (%); TC: total leucocyte count (cells/μl); N: neutrophils (%); L: lymphocytes (%); E: eosinophils (%); platelets (cells/μL); ESR: erythrocyte sedimentation rate (mm/HR); Fe: ferritin (ng/ml); CEA: carcinoembryonic antigen (ng/ml); LDH: lactate dehydrogenase (U/L); D-dimer (ng/mL); CRP: C-reactive protein (mg/dL); BMI: body mass index.

Time points	RBC	Hb	PCV	TC	N	L	E	Platelet	ESR	FE	CEA	LDH	D-dimer	CRP	Weight	BMI kg/m^2^
April 2024	5	14.6	43.1	6000	76.9	13.3	0.9	312000	20	48.5	2.57	154	220	2.7	55	21.8
May 2024	4.96	14.7	43.9	6110	59.3	32.1	1.4	265000	6	45	2.15	225	146	0.6	55.5	22

**Table 3:** Changes in safety profiles across time points. TB: total bilirubin (mg/dL); SGOT: serum glutamic oxaloacetic transaminase (U/L); SGPT: serum glutamic pyruvic transaminase (U/L); ALP: alkaline phosphatase (U/L); HbA1c: glycosylated hemoglobin (%); urea (mg/dL); creatinine (mg/dL); EGFR: estimated glomerular filtration rate (mL/min/1.73m^2^); uric acid (mg/dL); Na: sodium (mEq/L); K: potassium (mEq/L); Vit D: vitamin D-25-hydroxy vitamin D (ng/ml).

Time points	Liver function						HBA1C	Renal function								Vit D
	TB	SGOT	SGPT	ALP	Total protein	Albumin		Urea	Creatinine	eGFR	Uric acid	Na	K	HCO_3_	Cl	
April 2024	0.53	11.84	8.61	66.1	6.79	4.20	5.7	13.2	0.54	110	3.34	135	4.1	34.6	101	30
May 2024	0.57	11.79	9.21	61.6	7.03	4.22	5.7	7.13	0.55	109	3.94	137	4.6	35.1	105	29.4

**Table 4:** Changes in quality of life, symptoms, and survival rate scores. EORTC QLQ-C30: European Organization for Research and Treatment of Cancer Quality of Life Questionnaire.

Edmonton Symptom Assessment Scale									EORTC QLQ-C30 Scale			Chuang Survival Score
Nausea	Depression	Anxiety	Drowsy	Appetite	Well-being	Breathlessness	Pain	Tiredness	Global score	Functional score	Symptom score	1.5
0	0	9	8	5	6	0	0	0	58.3	73.3	25.6	
0	0	0	0	0	0	0	0	0	83.3	91.1	0	0

## Discussion

This case highlights the potential benefits of integrative oncology approaches in the management of high-grade gliomas. The patient experienced significant improvements following a comprehensive treatment protocol that included oncothermia and various complementary therapies.

Oncothermia, a form of modulated electro-hyperthermia, has been explored as an adjunctive treatment for various types of cancer, including high-grade gliomas [6]. Studies reported that oncothermia, in combination with chemotherapy and radiotherapy or as a monotherapy, improved tumor control and survival outcomes in patients with advanced cancers [7,8]. In this case, the patient's lesion size was reduced by approximately 12% following oncothermia sessions, indicating a positive response similar to findings in the existing literature.

High-dose vitamin C therapy has been investigated for its potential anticancer properties, including its role in enhancing the effectiveness of other treatments and reducing side effects. A recent review by Renner et al. suggests that high-dose intravenous vitamin C could enhance the cytotoxic effects of chemotherapy in glioblastoma cells. Furthermore, it will selectively induce oxidative stress and induce cytotoxicity in malignant cells [9]. Additionally, Ma et al. suggested that high-dose vitamin C could reduce inflammation and improve the quality of life in cancer patients [10]. The significant improvement in hematological parameters and mental health indices observed in our patient aligns with these findings.

Hydrogen inhalation therapy is an emerging complementary treatment in oncology, known for its antioxidant and anti-inflammatory effects. Previous studies have demonstrated that hydrogen gas can protect cells from oxidative stress and may improve outcomes in various conditions, including cancer [10]. A single case report in 2019 reported complete remission of brain tumor post hydrogen inhalation in a 44-year-old woman [11]. Our patient's improved clinical status and reduced lesion size support the potential benefits of hydrogen inhalation therapy.

Ozone therapy has been explored for its potential benefits in oncology, primarily due to its immunomodulatory and oxidative stress-reducing properties. Hypoxia is considered the hallmark of brain tumors and is associated with tumor progression, as it inhibits antitumor immune responses [12]. Luongo et al. reported that ozone therapy could enhance the effects of traditional cancer treatments by modulating the immune response and reducing tumor hypoxia [13]. Our patient included ozone therapy as part of the integrative protocol, contributing to the observed clinical improvements.

Diet therapy was another inclusion in this integrative protocol. The patient was advised to follow a low-carbohydrate diet in a time-restricted manner, ensuring daily fasting for 14 hours. Preclinical studies have shown that time-restricted feeding can slow down tumor growth. This effect is thought to be due to metabolic stress imposed on cancer cells [14], which have a high metabolic demand and are less adaptable to fasting conditions. Acupuncture is widely recognized for its ability to manage pain, reduce stress, and improve overall quality of life in cancer patients. An earlier case report suggested the role of acupuncture in regressing the size of oligodendroglioma in a 54-year-old man [15]. In the current study, we employed acupuncture to reduce headaches, improve cognitive functions, and promote general well-being. Studies suggest that acupuncture may benefit cancer patients by modulating the nervous system to relieve pain, enhancing the immune system, reducing inflammation, improving blood flow, regulating the endocrine system, supporting gastrointestinal function, and enhancing overall quality of life [16].

Magnet therapy is another complementary approach that has been explored for its potential to alleviate symptoms and improve the quality of life in cancer patients. Pulsed electromagnetic field (PEMF) therapy, an advanced form of magnet therapy, has demonstrated beneficial effects in oncology by inhibiting cell proliferation, reducing neovascularization, and exerting cytotoxic effects on cancer cells [17]. Our patient included PEMF therapy in his integrative treatment plan, which may have contributed to his overall clinical improvement. Hydrotherapy measures, such as hot foot baths and enemas, were given to our patient. These naturopathic remedies were provided to relieve fatigue and upregulate gastrointestinal function. Earlier studies have reported the usefulness of hot foot baths in relieving fatigue and improving the quality of life in cancer patients [18]. Our patients reported improvement in energy levels and relief of symptoms, which may be attributed to these therapies. Studies suggest that hydrotherapy may benefit cancer patients by relieving pain, reducing inflammation, enhancing blood circulation, stimulating the immune system, reducing stress, improving emotional well-being, and enhancing sleep quality [19].

Supplemental injections and therapies were also integrated into the patient's regimen. Dexamethasone and N-acetyl cysteine were administered to reduce brain swelling and improve cognitive function. Vitamin D and coenzyme Q10 were included to alleviate mitochondrial dysfunction and reduce fatigue, while Orokinase was used to lower D-dimer levels, addressing hypercoagulability often observed in cancer patients. Additionally, sublingual cannabis and melatonin (5 mg/day) were provided to enhance sleep quality. This comprehensive supplement strategy aimed to target multiple facets of the patient’s condition, contributing to an overall improvement in health status. Hydrosun therapy, also known as hydrotherapy combined with infrared radiation, has garnered attention for its potential therapeutic benefits in various medical conditions, including oncology [20]. In our case, the patient reported significant improvements in energy levels and overall symptom relief following Hydrosun therapy sessions.

The reported outcomes suggest that the combination of these complementary medicine therapies may have synergistic effects, leading to tumor size regression, significant symptomatic relief, and enhanced quality of life for cancer patients. This may be considered the ability of integrative oncological approaches to induce spontaneous regression, suggesting the reversal of cancer cells into normal cells without any conventional therapies [21]. However, it is crucial to note that this case study represents a single patient's experience, and larger, controlled studies are necessary to establish the efficacy and safety of such integrative approaches in oncology. Additionally, the patient's psychological state and potential placebo effects may have influenced the outcomes. Another limitation of this case report is the difficulty in identifying the individual effects of each therapy included in the integrative treatment protocol. While the overall improvement in the patient’s condition is promising, it is challenging to attribute specific outcomes to any single therapy due to the synergistic and cumulative effects of the combined treatments. It is also important to note that this is a preliminary finding, and hence cannot be considered conclusive. Nevertheless, the patient was happy that he was asymptomatic and had resumed his day-to-day activities as before.

## Conclusions

In clinical practice, integrative oncology often involves multiple modalities used concurrently, making it complex to isolate the impact of each therapy. This integrative approach, while potentially more effective, complicates the ability to conduct a clear analysis of the efficacy of individual components. Future research should focus on the mechanistic understanding of how these therapies interact and their long-term impact on cancer progression and patient well-being.
